# Integrated Volatile Metabolome and Transcriptome Analysis Provides Insights into Floral Aroma Biosynthesis in Waterlilies (*Nymphaea* L.)

**DOI:** 10.3390/plants15030384

**Published:** 2026-01-27

**Authors:** Qun Su, Fengshun Wang, Jiahui Zhao, Jianxun Lu, Hongyan Wang, Yanfei La, Zhenglin Wan, Yuling Lin, Min Tian, Lingyun Wang, Zhongxiong Lai

**Affiliations:** 1 Institute of Horticultural Biotechnology, Fujian Agriculture and Forestry University, Fuzhou 350002, China; qunsu315@yeah.net (Q.S.); buliang84@163.com (Y.L.); 2 Flower Research Institute, Guangxi Academy of Agricultural Sciences, Nanning 530007, China; wangfengshun@126.com (F.W.); zhaojiahui2213@163.com (J.Z.); 18154591713@163.com (J.L.); wanghongyan721@126.com (H.W.); layanfei1109@163.com (Y.L.); wanzhenglin0700227@163.com (Z.W.); 3 Key Laboratory for Flower Breeding of Yunnan Province, Floriculture Research Institute, Yunnan Academy of Agricultural Sciences, National Engineering Research Center for Ornamental Horticulture, Kunming 650200, China; 4 Laboratory of Characteristic Aquatic Vegetable Breeding and Cultivation, Jinhua Academy of Agricultural Sciences, Jinhua 321000, China

**Keywords:** *Nymphaea* ‘Paul Stetson’ stamen, volatile organic compound, floral aroma, terpenoid biosynthesis, volatile metabolome and transcriptome analysis

## Abstract

Waterlily (*Nymphaea* L.), a globally renowned aquatic ornamental plant, is prized for its aesthetic flowers and intense floral fragrance. However, the molecular mechanisms underlying floral scent biosynthesis in waterlily remain poorly characterized, and integrated analyses of dynamic volatile emission patterns and their associated biosynthetic pathways are lacking. In this study, we combined headspace solid-phase microextraction/gas chromatography–mass spectrometry (HS-SPME/GC-MS) with transcriptome sequencing (RNA-seq) to investigate the composition, emission dynamics, and biosynthesis of volatile organic compounds (VOCs) in the stamens of *Nymphaea* ‘Paul Stetson’ across three developmental stages. A total of 671 VOCs, classified into 14 categories, were identified. Transcriptome analysis revealed 47,951 differentially expressed genes (DEGs). Integrative omics analysis demonstrated correlated DEGs and differentially accumulated volatiles were significantly enriched in pathways related to phenylpropanoid biosynthesis, terpenoid backbone biosynthesis, diterpenoid biosynthesis, and ubiquinone/other terpenoid-quinone biosynthesis. Five candidate functional genes exhibiting strong positive correlations with VOC accumulation levels were identified, three of which are implicated in terpenoid biosynthesis. These findings provide a theoretical foundation for elucidating aroma composition and biosynthesis in waterlily and offer novel avenues for the genetic improvement of fragrance traits for ornamental, beverage, and cosmetic applications.

## 1. Introduction

Plant floral volatile compounds comprise a diverse array of low-molecular-weight, low-boiling-point, and highly volatile substances released from flowers. These volatiles play crucial roles in attracting insect pollinators and providing defense against predators and pathogens [1,2]. Beyond floral organs, certain vegetative tissues of plants can also emit aromatic compounds. The emission levels of floral scent vary significantly depending on the developmental stage of the flower [3]. For many ornamental plants, floral fragrance constitutes a key ornamental trait. To date, over 1700 volatile compounds have been identified from more than 1000 flower species. These volatiles are primarily categorized into three major classes: terpenoids, phenylpropanoids/benzenoid compounds, and fatty acid derivatives, with terpenoids representing the most predominant and diverse group [2,4,5]. Compared to readily observable plant traits such as flower morphology and color, research on floral fragrance has received comparatively less attention [6].

With the rapid advancement of technology, significant progress has been made in the techniques for separating and identifying floral scent components, as well as in understanding their molecular regulatory mechanisms. Integrated volatile metabolomics and transcriptome sequencing have emerged as crucial approaches in recent years for investigating the composition and molecular biosynthesis mechanisms of plant floral fragrances. Li et al. [7] conducted a comparative analysis of two *Lonicera* cultivars, ‘Yujin2’ (strong aroma) and ‘Fengjin1’ (bland odor). They identified that the biosynthesis pathways of terpenoids (monoterpenoids, including geraniol and alpha-terpineol; sesquiterpenoids, including farnesol, farnesal, and alpha-farnesene; triterpenoid squalene), tryptophan and its derivatives (methyl anthranilate), and fatty acid derivatives were the primary contributors to the stronger aroma of ‘Yujin2’ compared to ‘Fengjin1’. Furthermore, they analyzed the expression patterns of key structural genes within the relevant volatile organic compound (VOC) biosynthesis pathways. Wang et al. [8] employed gas chromatography–mass spectrometry (GC-MS) to analyze the volatile profiles in petals of two *Prunus mume* cultivars with markedly distinct aromas, ‘Xiao Lve’ and ‘Xiangxue Gongfen’, across different flowering stages. A total of 44 volatile compounds were detected, with eugenol, cinnamyl acetate, hexyl acetate, and benzyl acetate identified as the principal constituents responsible for the characteristic *Prunus mume* scent. By integrating transcriptome sequencing data, they constructed regulatory networks for the biosynthesis of key aroma compounds in both cultivars. Zhu et al. [9] investigated the volatile profiles and associated genes in flowers of *Chrysanthemum indicum* var. aromaticum across three distinct developmental stages. They identified 370 volatile metabolites, predominantly terpenoids and esters, and discovered that key differentially expressed genes (DEGs) involved in volatile terpene biosynthesis were specifically identified within the MEP and its downstream pathways. Luo et al. [10] detected 22 VOCs in flowers of *Rosa chinensis* ‘Old Blush’ (‘OB’) and *R. chinensis* ‘Chilong Hanzhu’ (‘CH’). The predominant VOC in ‘OB’ petals was identified as 1,3,5-trimethoxybenzene (TMB), whereas 2-phenylethanol (2-PE) was the major component in ‘CH’. Furthermore, 15 key genes implicated in phenylpropanoid biosynthesis were identified. The function of *RcCH_AADC-1* was subsequently validated through transgenic experiments in tobacco. Collectively, these studies demonstrate that integrated volatile metabolomic and transcriptomic analysis plays a crucial role in elucidating the composition and molecular biosynthesis mechanisms of plant floral fragrance.

Waterlilies (*Nymphaea* L.) within the family Nymphaeaceae are globally renowned aquatic ornamentals, prized for their captivating floral colors and elegant, enchanting fragrance. Often celebrated as the ‘sleeping beauty among flowers’ and the ‘living palette of ponds’ they hold significant popularity worldwide. Among the five subgenera of *Nymphaea*, the sub. *Brachyceras* exhibits the most intense floral scent, demonstrating considerable potential for development and application in essential oils, perfumery, skincare products, food processing, and pharmaceuticals [11,12,13,14]. While substantial research has detailed the floral fragrance of plants such as *Rosa chinensis* [10,15,16], *Lilium* [17,18,19], *Dendranthema morifolium* [9,20,21], *Nelumbo nucifera* [22,23,24], and *Prunus mume* [8,25,26], studies on waterlily scent remain in their nascent stages. Current research is relatively limited and primarily focuses on: (1) the identification and classification of VOCs in flowers of different *Nymphaea* cultivars, or different parts and flowering stages of the same cultivar [27,28,29,30,31]; and (2) the analysis of waterlily VOCs and their relationship with putative pollinators [32,33]. Consequently, the molecular mechanisms underlying waterlily fragrance biosynthesis are poorly understood, and integrated analyses of dynamical aromatic release patterns and the associated internal molecular synthesis pathways are notably lacking [34].

*Nymphaea* ‘Paul Stetson’ represents an important and distinctive cultivar within the sub. *Brachyceras*. Characterized by prolific flowering, robust and erect peduncles, and an intense floral fragrance, it possesses high ornamental value. Beyond its aesthetic appeal, the flowers can be processed into herbal tea and exhibit considerable potential for development in essential oils and cosmetics. Furthermore, the leaves of *Nymphaea* ‘Paul Stetson’ exhibit a unique viviparous developmental characteristic, enabling rapid propagation of large numbers of individuals within a short timeframe [35]. Consequently, its seedlings are relatively inexpensive, offering significant value for application and widespread adoption. This cultivar is now extensively cultivated in southern China.

Previous studies indicate that VOC extraction yield is highest on the first day of flowering, with stamens being the primary emission site, accounting for 70–90% of total floral volatiles, and showing no significant difference from whole-flower VOC content [27,30,36]. Therefore, stamen VOC profiles reliably represent the whole flower. In this study, we employed HS-SPME/GC-MS and RNA-seq to identify and analyze VOCs in the stamens of *N*. ‘Paul Stetson’ across three developmental stages. Our findings provide foundational data for elucidating VOC formation mechanisms and establish a basis for quality breeding within *Nymphaea*.

## 2. Results

### 2.1. Volatile Organic Compound Profiling of Nymphaea Stamens Across Developmental Stages

**Figure 1:**
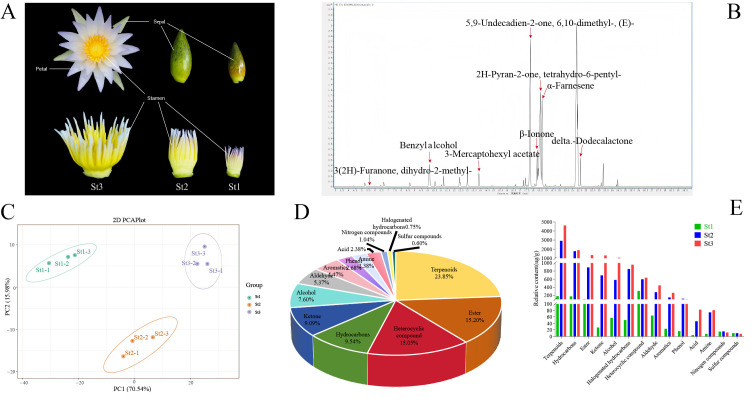
GC–MS profiling of volatile constituents in *Nymphaea* ‘Paul Stetson’ stamens across developmental phases. (**A**) Phenotypic progression of floral stamens during development. (**B**) Representative total ion chromatograms of stamen volatile profiles. (**C**) Principal component analysis (PCA) of all nine biological replicates. (**D**) Compositional distribution of detected volatile organic compounds (VOCs). (**E**) Comparative abundance of major VOC classes across developmental stages.

To characterize the dynamic composition of floral scent from bud to bloom, VOCs in stamens in three different developmental phases (St1: small bud, St2: large bud, St3: anthesis) were analyzed by HS-SPME-GC-MS (Figure 1A,B). Principal component analysis (PCA) revealed significant compositional divergence among the three developmental stages, with principal components 1 and 2 accounting for 70.54% and 15.98% of total variance, respectively (Figure 1C). A total of 671 VOCs were identified across all stages, categorized into 14 chemical classes (Supplementary Table S1, Figure S1). Terpenoids (160 compounds), esters (102), heterocyclic compounds (101), hydrocarbons (64), ketones (61), and alcohols (51) constituted the predominant VOC groups, representing 23.85%, 15.20%, 15.05%, 9.54%, 9.09%, and 7.60% of total volatiles, respectively. The remaining 132 VOCs were distributed across eight minor classes: aldehydes (36; 5.37%), aromatics (30; 4.47%), phenols (18; 2.68%), amines (16; 2.38%), acids (16; 2.38%), nitrogen compounds (7; 1.04%), halogenated hydrocarbons (5; 0.75%), and sulfur compounds (4; 0.60%) (Figure 1D).

Across all samples, 671 VOCs were identified, with stage-specific counts of 531 (St1), 673 (St2), and 671 (St3) compounds. Terpenoids constituted the most abundant chemical class. The quantities of esters, heterocyclic compounds, hydrocarbons, ketones, alcohols, aldehydes, aromatics, and phenols consistently decreased across successive developmental stages. While St2 and St3 exhibited comparable total VOC counts and minimal inter-stage variation in category-specific numbers relative to St1 (Supplementary Table S2), the significantly enhanced fragrance intensity in St3 primarily resulted from differential accumulation of key volatiles. Terpenoids, esters, heterocyclic compounds, hydrocarbons, ketones, alcohols, and aldehydes represented the dominant VOC categories across all three stages. Relative abundance analysis revealed distinct accumulation patterns (Figure 1E). Most VOC classes exhibited progressively increasing concentrations during stamen development, with substantial accumulation occurring at St2 and peaking at St3. For instance, the mean concentrations of α-farnesene and humulene were only 0.08 and 2.12, respectively, at stage St1, increased markedly to 28.51 and 511.92 at stage St2, and finally peaked at 40.85 and 940.50 by stage St3. Among all classes, terpenoids reached maximal accumulation at St3 (4616.79 μg/g), representing a 57.8% increase from St2 (2925.68 μg/g). Hydrocarbons showed secondary abundance (1893.37 μg/g at St3 vs. 1782.15 μg/g at St2). Esters increased 52.3% from St2 (891.74 μg/g) to St3 (1358.28 μg/g). Ketones demonstrated 88.5% growth (692.18 μg/g at St2 vs. 1305.02 μg/g at St3). Notably elevated abundances at St3 were also observed for: alcohols: 1072.19 μg/g; halogenated hydrocarbons: 955.76 μg/g; heterocyclic compounds: 633.67 μg/g; aldehydes: 444.96 μg/g; aromatics: 258.94 μg/g; phenols: 103.32 μg/g. This accumulation profile indicates these compound classes critically contribute to waterlily scent emission (Supplementary Table S3).

### 2.2. Comparative Analysis of Volatile Organic Compounds in Nymphaea Stamens Across Developmental Stages

**Figure 2:**
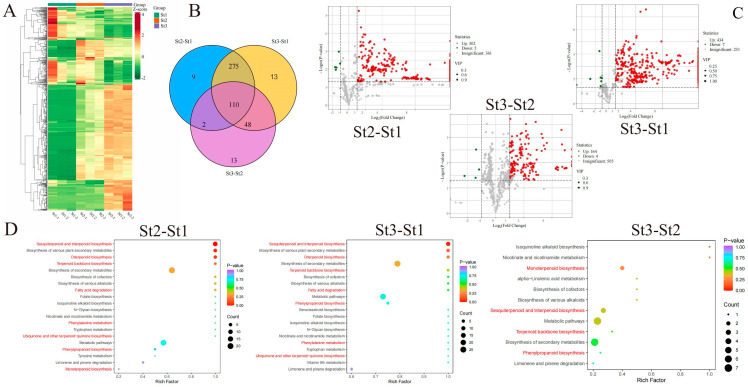
Comparative profiling of volatile organic compounds (VOCs) in *Nymphaea* ‘Paul Stetson’ stamens across developmental stages. (**A**) Heatmap visualization of dynamic VOC accumulation patterns. (**B**) Venn diagram representation of differentially accumulated volatiles (DAVs). (**C**) Volcano plots identifying statistically significant DAVs. (**D**) Functional enrichment analysis of KEGG pathways associated with DAVs in pairwise comparisons.

Clustering analysis revealed distinct accumulation patterns for VOCs in waterlily stamens during both bud and flowering stages. Most differentially accumulated volatiles (DAVs) exhibited significant enrichment specifically at bud stage St2 and anthesis stage St3 (Figure 2A). Among 470 DAVs identified across all three stages, seven accumulation patterns were discerned (Figure S2). Pairwise comparisons demonstrated: St2 vs. St1: 396 DAVs (390 upregulated, 6 downregulated); St3 vs. St1: 446 DAVs (435 upregulated, 11 downregulated); St3 vs. St2: 173 DAVs (167 upregulated, 6 downregulated). Notably, 110 DAVs were conserved across all developmental phases, potentially harboring key constituents for floral scent formation (Figure 2B, Supplementary Table S4). Volcano plots further delineated stage-specific dynamics: St2 vs. St1: 302 VOCs upregulated vs. 5 downregulated; St3 vs. St1: 434 VOCs upregulated vs. 7 downregulated; St3 vs. St2: minimal divergence (164 differential VOCs). This progression indicates continuous VOC accumulation during stamen maturation, peaking at anthesis (St3) with maximal scent emission (Figure 2C). KEGG enrichment analysis revealed DAVs predominantly associated with VOC biosynthetic pathways: sesquiterpenoid and triterpenoid biosynthesis, diterpenoid biosynthesis, terpenoid backbone biosynthesis, fatty acid degradation, phenylpropanoid biosynthesis, phenylalanine metabolism, and ubiquinone/terpenoid-quinone biosynthesis (Figure 2D).

### 2.3. Identification of Key Aromatic Volatiles in Nymphaea Stamens Across Developmental Stages

**Figure 3:**
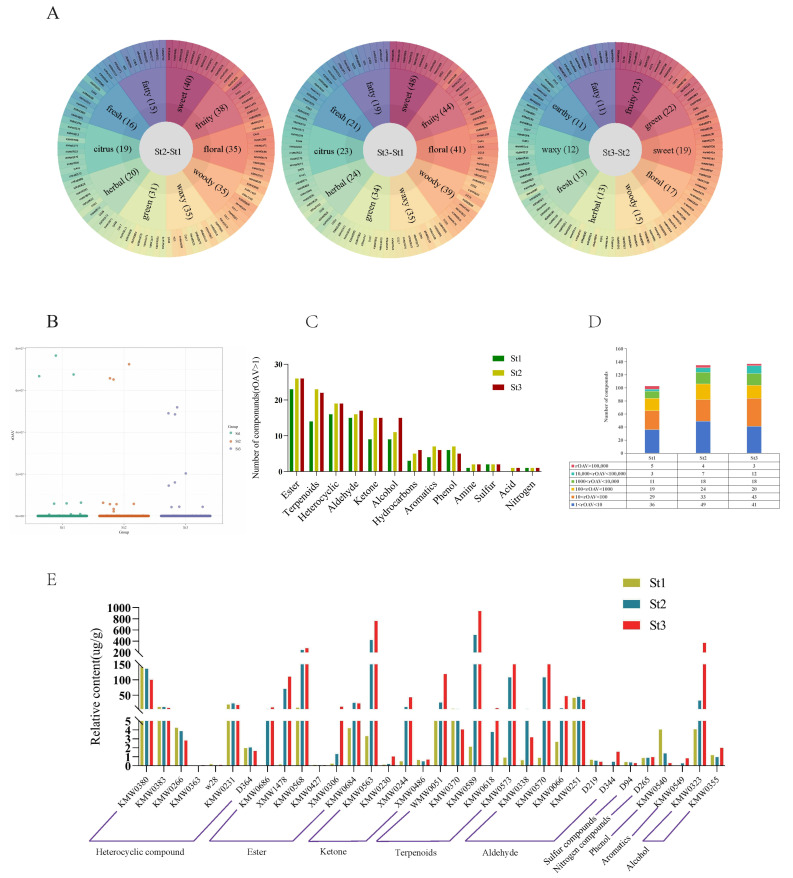
Identification of key odor-active compounds in *Nymphaea* ‘Paul Stetson’ stamens across developmental stages. (**A**) Molecular odor wheel visualizing odor-active volatiles (rOAV-based) for each comparison group. (**B**) Scatter plot distribution of relative odor activity values (rOAVs) for volatile organic compounds. (**C**) Quantitative analysis of volatiles exhibiting rOAV > 1 in stamen samples per developmental group. (**D**) Frequency distribution of compounds with rOAV > 1 across stamen developmental stages. (**E**) Temporal accumulation patterns of individual volatiles demonstrating rOAV > 1000 during development.

Aroma profiles objectively characterize sensory attributes among samples. Sensory evaluation of DAVs revealed distinct olfactory signatures. As shown in Figure 3A, the top five sensory descriptors for DAVs in St2 vs. St1 and St3 vs. St1 comparisons were sweet, fruity, floral, woody, and waxy. Conversely, St3 vs. St2 DAVs exhibited fruity, green, sweet, floral, and woody as predominant notes (Figure S3). To pinpoint aroma-active compounds, relative odor activity values (rOAVs) were calculated for stamen volatiles. Volatiles with rOAV > 1 were established as direct contributors to waterlily scent [37]. Our analysis identified: St1: 103 volatiles with rOAV > 1, St2: 135 volatiles with rOAV > 1, St3: 137 volatiles with rOAV > 1. Notably, rOAV magnitudes increased substantially during St2 and St3 (Figure 3B, Supplementary Table S5), indicating these stages govern intense floral fragrance production and release. These aroma-active compounds comprised 13 chemical classes, with esters representing the largest group across all developmental phases, followed by terpenoids, heterocyclic compounds, aldehydes, and ketones (Figure 3C, Supplementary Table S5).

Statistical analysis of rOAV distributions revealed distinct patterns across stages: St1 vs. St2: VOCs with rOAV 1–10 predominated numerically. St3: VOCs exhibiting rOAV 10–100 constituted the largest proportion (Figure 3D). Thirty-four volatiles with rOAV ≥ 1000 were identified as primary odorants, detected in at least one developmental phase. Stage-specific occurrences were: St1: 19 compounds; St2: 29 compounds; St3: 33 compounds. These high-impact volatiles comprised: heterocyclic compounds (7), esters (6), aldehydes (6), terpenoids (5), ketones (4), alcohols (2). There were single representatives from sulfur compounds, nitrogen compounds, phenols, and aromatics (Figure 3E, Supplementary Table S6). Notably, nine high-rOAV VOCs demonstrated significant accumulation in St2/St3 stamens, substantially exceeding other volatiles and peaking predominantly at St3: humulene, (E)-5,9-undecadien-2-one, 6,10-dimethyl-, benzyl alcohol, tetrahydro-6-pentyl-2H-pyran-2-one, (E)-β-farnesene, (E)-2-dodecenal, β-ionone, geranyl isobutyrate, and 2-methoxy-3-(2-methylpropyl)pyrazine. These compounds represent key contributors to enhanced floral fragrance during the bud-to-bloom transition (Figure 3E, Supplementary Table S6).

### 2.4. Transcriptomic Profiling of Nymphaea Stamens Across Developmental Stages

**Figure 4:**
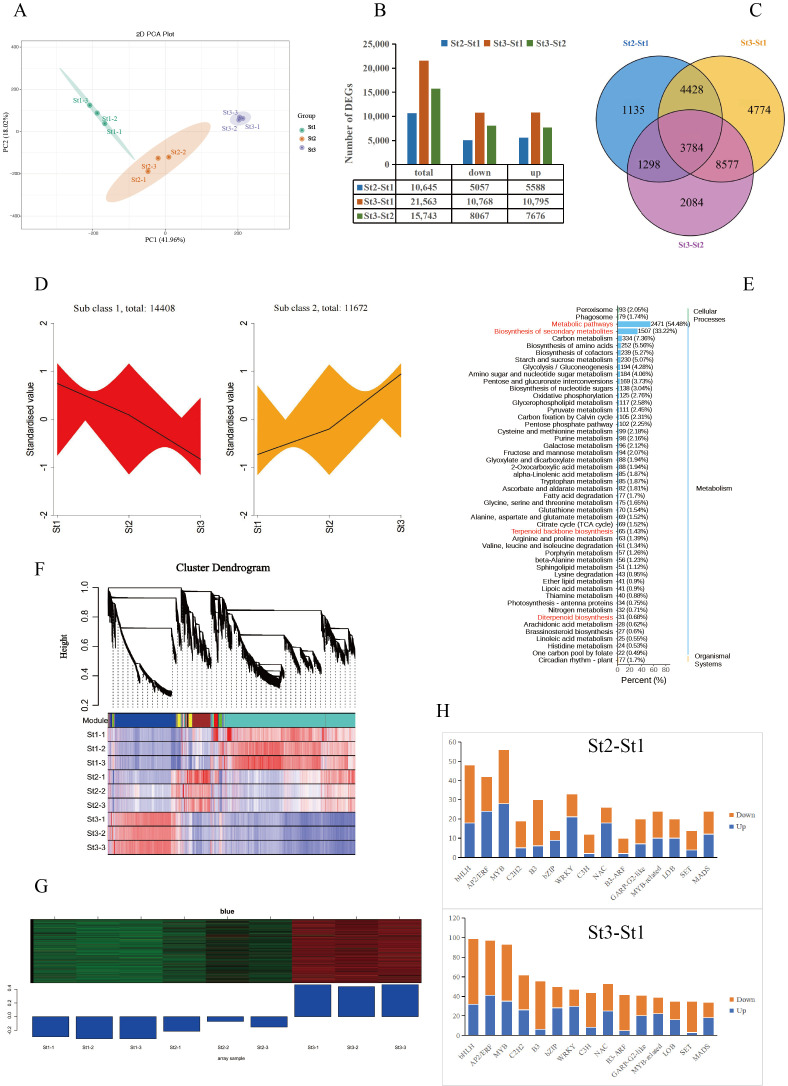
Functional characterization of differentially expressed genes (DEGs) in *Nymphaea* ‘Paul Stetson’ stamens across developmental stages. (**A**) Principal component analysis (PCA) of stamen transcriptomes. (**B**) Quantitative distribution of DEGs during stamen development. (**C**) Venn diagram illustrating overlapping DEGs. (**D**) K-means clustering of 26,080 DEGs. (**E**) Twenty top-ranked enriched KEGG pathways for DEGs in each comparison group. (**F**) Hierarchical clustering of co-expression modules derived from WGCNA. (**G**) Expression dynamics of DEGs within the ‘blue’ co-expression module. (**H**) Top 15 differentially expressed transcription factors.

To elucidate the molecular basis of volatile metabolism during floral scent formation from bud to anthesis, we conducted transcriptome sequencing of stamens at stages St1, St2, and St3. Nine libraries generated 69.36 GB of clean reads, with average values for Q20, Q30, and GC content of 97.68%, 93.44%, and 48.94%, respectively, confirming high sequencing accuracy suitable for downstream analysis (Supplementary Table S7). Principal component analysis revealed effective sample segregation into three developmentally distinct clusters (PC1: 41.96% variance; PC2: 18.02%), demonstrating high intra-stage reproducibility and inter-stage divergence (Figure 4A). Pairwise comparisons identified 47,951 differentially expressed genes (DEGs): St2 vs. St1: 10,645 DEGs (5057 up; 5588 down); St3 vs. St1: 21,563 DEGs (10,768 up; 10,795 down); St3 vs. St2: 15,743 DEGs (8067 up; 7676 down). Notably, 3784 DEGs were conserved across all comparisons, with near-balanced up/downregulation observed in each contrast (Figure 4B,C; Supplementary Table S8). K-means clustering partitioned 26,080 DEGs into two expression profiles: Cluster 1: 14,408 DEGs, Cluster 2: 11,672 DEGs (exhibiting expression patterns congruent with scent metabolite accumulation, suggesting regulatory roles in fragrance biosynthesis) (Figure 4D). Functional annotation of Cluster 2 DEGs against KEGG revealed significant enrichment in metabolic pathways, secondary metabolite biosynthesis, terpenoid backbone biosynthesis, and diterpenoid biosynthesis. Additionally, there were VOC-associated pathways including phenylpropanoid biosynthesis and carotenoid biosynthesis and complementary routes including fatty acid biosynthesis, flavonoid biosynthesis, amino acid biosynthesis, and monoterpenoid biosynthesis (Figure 4E).

To decipher regulatory mechanisms underlying VOC biosynthesis in waterlily, we employed weighted gene co-expression network analysis (WGCNA), identifying 16 distinct gene modules through systematic clustering (Figure 4F). Significant differential expression patterns were observed across modules, with the ‘blue’ module (containing 9143 DEGs) demonstrating strong correlation with VOC accumulation from bud to anthesis. This module exhibited minimal expression at St1, followed by progressive upregulation peaking at St3 (Figure 4G). Transcriptional factor (TF) profiling revealed 751, 1538, and 1044 differentially expressed TFs in St2 vs. St1, St3 vs. St1, and St3 vs. St2 comparisons, respectively. The bHLH, AP2/ERF, MYB, C2H2, B3, bZIP, and WRKY families constituted the predominant TF groups across all pairwise comparisons. Focusing on the top 15 TF families including St2 vs. St1, MYB predominated (28 upregulated, 28 downregulated), followed by bHLH (18 up, 30 down), AP2/ERF (24 up, 18 down), WRKY (21 up, 12 down), B3 (6 up, 24 down), NAC (18 up, 8 down), and MYB-related (10 up, 14 down); St3 vs. St1: bHLH represented the most abundant family (32 up, 67 down), succeeded by AP2/ERF (41 up, 56 down), MYB (35 up, 58 down), C2H2 (26 up, 36 down), B3 (6 up, 50 down), bZIP (28 up, 22 down), and WRKY (30 up, 17 down) (Figure 4H).

### 2.5. Analysis of Terpenoid- and Phenylpropanoid-Biosynthesis-Related Genes

**Figure 5:**
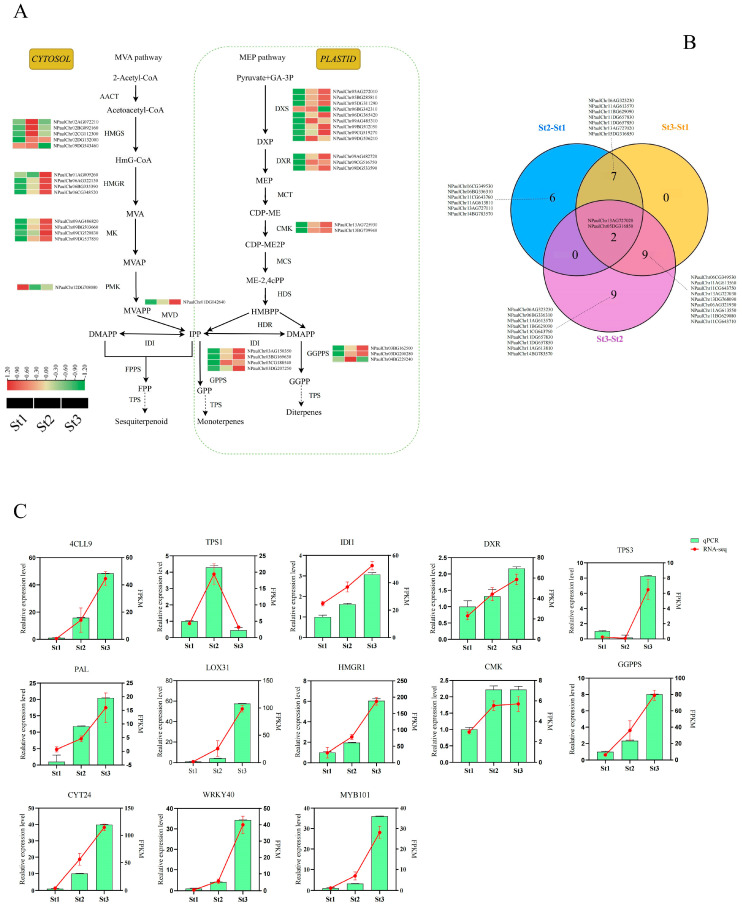
Expression profiles of key genes encoding enzymes implicated in terpenoid biosynthesis. (**A**) Schematic of terpenoid biosynthetic pathways (MVA and MEP) in Nymphaea. Key enzymatic components include: hydroxymethylglutaryl-CoA synthase (HMGS), 3-hydroxy-3-methylglutaryl-CoA reductase (HMGR), mevalonate kinase (MK), phosphomevalonate kinase (PMK), diphosphomevalonate decarboxylase (MVD), 1-deoxy-D-xylulose-5-phosphate synthase (DXS), 1-deoxy-D-xylulose-5-phosphate reductoisomerase (DXR), 4-diphosphocytidyl-2-C-methyl-D-erythritol kinase (CMK), geranylgeranyl diphosphate synthase (GGPPS), and isopentenyl diphosphate Δ-isomerase (IDI). (**B**) Venn diagram representation of differentially expressed terpene synthase (TPS) genes. (**C**) qRT-PCR validation of gene expression level in the transcriptome. Data are shown as mean ± standard deviation of three biological replicates (*p*< 0.05).

Terpenoids constitute pivotal components of waterlily floral scent biosynthesis. In plants, terpenoid production occurs primarily through two distinct metabolic trajectories: (1) the cytosolic/mitochondrial mevalonate (MVA) pathway, generating secondary metabolites including sterols, sesquiterpenes, and triterpenes; and (2) the plastidial methylerythritol phosphate (MEP) pathway, predominantly responsible for monoterpene, diterpene, and carotenoid biosynthesis [38,39]. To elucidate regulatory mechanisms underlying terpenoid accumulation during stamen development from bud to anthesis, we identified DEGs encoding terpenoid biosynthetic enzymes (Figure 5A, Supplementary Table S9). Critical DEGs exhibiting upregulated expression predominately during St2 and St3 included: *NPaulChr02AG072210*, *NPaulChr02CG112300*, and *NPaulChr09DG543460* (hydroxymethylglutaryl-CoA synthase), *NPaulChr06AG322130*, *NPaulChr06BG335390*, and *NPaulChr06CG348520* (3-hydroxy-3-methylglutaryl-coenzyme A reductase), *NPaulChr05DG311290* (1-deoxyxylulose-5-phosphate synthase), *NPaulChr09AG482720* (1-deoxyxylulose-5-phosphate reductoisomerase), *NPaulChr03BG162500* and *NPaulChr03DG200280* (2E,6E-farnesyl diphosphate synthase), *NPaulChr03BG169630* (isopentenyl diphosphate isomerase). Notably, maximal expression levels for most genes occurred at stage St3.

Terpene synthases (TPSs), catalyzing the terminal step in terpenoid biosynthesis, constitute rate-limiting catalysts in floral scent formation. Among 23 differentially expressed TPS genes identified across pairwise stamen comparisons, stage-specific distributions were observed: 15 in St2 vs. St1, 18 in St3 vs. St1, and 20 in St3 vs. St2. Two conserved TPS genes—*NPaulChr13AG727020* (terpene synthase 13) and *NPaulChr05DG316850* (ent-kaurene synthase B)—exhibited differential expression across all comparisons, potentially serving as key regulators of terpenoid-derived fragrance compounds (Figure 5B). Expression profiling revealed distinct temporal patterns: 5 TPS genes peaked at St1 with progressively diminished expression, 11 TPS genes (including *NPaulChr06AG323230*, *NPaulChr06BG336310*, *NPaulChr06CG349530*, *NPaulChr11AG613570*, *NPaulChr11BG629090*) showed maximal expression at St2, 7 TPS genes (*NPaulChr13AG727020*, *NPaulChr11CG643750*, *NPaulChr11AG613550*, *NPaulChr11CG643710*, *NPaulChr13DG768090*, *NPaulChr11AG613560*, *NPaulChr13AG727030*) reached peak expression at St3. This spatiotemporal expression aligns precisely with developmental VOC accumulation patterns (Figure S4). Tissue-specific analysis further demonstrated that 11 TPS genes (notably *NPaulChr13AG727020* and *NPaulChr11AG613570*) exhibited stamen-predominant expression, with secondary expression in petals/pistils (Figure S5). Collectively, these TPS candidates likely modulate terpenoid flux to enhance floral scent emission during anthesis.

KEGG enrichment analysis revealed consistent, significant enrichment of phenylpropanoid biosynthesis pathways across all comparison groups. Consequently, we examined the expression profiles of 68 differentially expressed genes (DEGs) encoding enzymes in this pathway across three developmental stages. Most DEGs demonstrated peak expression at St1, followed by intermediate levels at St2, with minimal expression observed at St3. This pattern indicates an overall declining trajectory of phenylpropanoid-related gene expression during stamen maturation. Notably, key enzymatic genes, including *NPaulChr10DG597040* (encoding *C4H*) and *NPaulChr02BG090410* (encoding *4CL*), exhibited maximal transcript abundance specifically at St3 (Figure S6, Supplementary Table S10). These contrasting expression dynamics suggest complex regulatory roles for phenylpropanoid biosynthetic enzymes in waterlily volatile compound synthesis.

To independently assess the quality of our transcriptome sequencing results, we randomly selected 13 genes encoding key enzymes (including *4CLL9*, *DXR*, *HMGR*, *IDI1*, *GGPPS*, and *TPS3*) for quantitative reverse transcription PCR analysis. The relative expression levels of these genes across stages St1, St2, and St3 demonstrated expression profiles congruent with transcript abundance patterns derived from RNA-seq FPKM values (Figure 5C). This concordance confirms the reliability and precision of our transcriptomic dataset.

### 2.6. Integration of Transcriptomic and Metabolomic Dynamics

**Figure 6:**
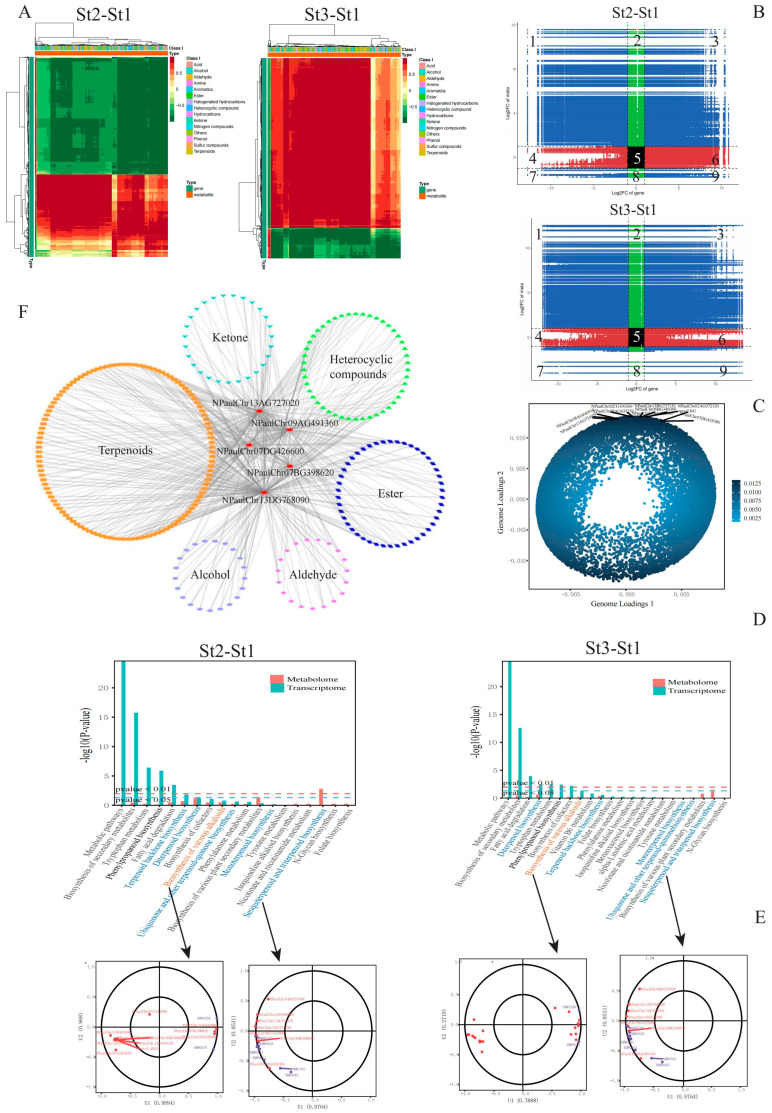
Integrated metabolomic and transcriptomic profiling of volatile organic compounds (VOCs) accumulated in *Nymphaea* ‘Paul Stetson’ stamens across developmental stages. (**A**) Hierarchical clustering heatmap illustrating correlations between differentially expressed genes (DEGs) and differentially accumulated volatiles (DAVs). (**B**) Nine-quadrant plot distributions of DEGs and DAVs across pairwise comparison groups. (**C**) Loading plot for DEGs, with blue dots denoting individual DEGs. (**D**) KEGG pathway enrichment analysis for the St2 vs. St1 and St3 vs. St1 comparisons. (**E**) Canonical correlation analysis of DEGs and DAVs associated with sesquiterpenoid/triterpenoid biosynthesis and alkaloid biosynthesis pathways in the St2 vs. St1 and St3 vs. St1 comparisons, respectively. DEGs and DAVs are represented by red and purple dots. (**F**) Co-expression network analysis between DEGs and DAVs, where red nodes indicate DEGs.

To elucidate regulatory mechanisms underlying volatile accumulation during stamen development from bud to anthesis, we performed correlation analysis between DEGs and DAVs. Pairwise comparisons revealed stage-specific associations: St2 vs. St1: 9442 DEGs correlated with 397 DAVs; St3 vs. St1: 19,966 DEGs correlated with 444 DAVs; St3 vs. St2: 14,265 DEGs correlated with 168 DAVs. Focusing on St2 vs. St1 and St3 vs. St1 comparisons for detailed analysis (Figure 6A), nine-quadrant plotting demonstrated positive DEG-DAV correlations primarily in quadrants 3/7, while negative correlations clustered in quadrants 1/9 (Figure 6B). Orthogonal two-way partial least squares (O2PLS) modeling further integrated transcriptomic (26,080 DEGs) and metabolomic (473 DAVs) datasets (Figure 6C). The ten DEGs exhibiting the strongest metabolomic covariation were *NPaulChr02CG100360* (hypothetical protein EJ110), *NPaulChr13BG737120* (senescence-specific cysteine protease), *NPaulChr06BG346040* (glycerol-3-phosphate acyltransferase 5), *NPaulChr08AG435250* (hypothetical protein EJ110), *NPaulChr02AG072120* (Δ^1^-pyrroline-2-carboxylate reductase), *novel.841* (hypothetical protein EJ110), *NPaulChr07DG425080* (CCCH-type zinc finger protein), *NPaulChr13AG724050* (non-specific lipid-transfer protein 3), *NPaulChr08AG440910* (proline-rich receptor-like kinase PERK3), and *NPaulChr09AG486380* (polyol transporter 5).

KEGG enrichment analysis of correlated DEGs and DAVs identified 19 significantly enriched pathways in the St2 vs. St1 comparison and 22 pathways in St3 vs. St1. Key enriched pathways included phenylpropanoid biosynthesis, terpenoid backbone biosynthesis, diterpenoid biosynthesis, ubiquinone and other terpenoid-quinone biosynthesis, monoterpenoid biosynthesis, and sesquiterpenoid/triterpenoid biosynthesis (Figure 6D). Canonical correlation analysis (CCA) further revealed robust covariation patterns between DEGs and DAVs across these metabolic routes (Figure 6E). Notably, five genes exhibited significant positive correlations (Pearson correlation coefficient > 0.8) with VOC accumulation, particularly terpenoids: *NPaulChr13DG768090*, *NPaulChr13AG727020* (encoding 3S,6E-nerolidol synthase, NES1), *NPaulChr07BG398620*, *NPaulChr07DG426600* (encoding cytochrome P450s), *NPaulChr09AG491360* (encoding farnesyl-diphosphate farnesyltransferase, SS1). Three of these genes participate directly in terpenoid biosynthetic pathways (Figure 6F, Supplementary Table S11).

### 2.7. Subcellular Localization and Transient Overexpression Analysis

**Figure 7:**
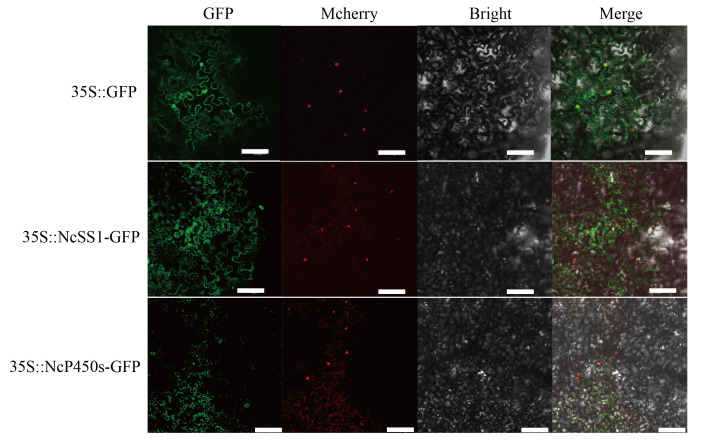
Subcellular localization of NcSS1 and NcP450s in tobacco leaves, bar = 100 μm.

In plants, monoterpenes are synthesized from GPP in plastids, whereas sesquiterpenes are produced from FPP in the cytoplasm. To clarify their functional sites within cells, we successfully cloned the CDS regions of NcP450s (*NPaulChr07BG398620*, involved in phenylpropanoid/aromatic compound biosynthesis) and NcSS1 (*NPaulChr09AG491360*, involved in terpenoid biosynthesis) and performed subcellular localization. The results showed that both NcP450s (*NPaulChr07BG398620*) and NcSS1 (*NPaulChr09AG491360*) exhibited a diffuse distribution within the cells (Figure 7).

**Figure 8:**
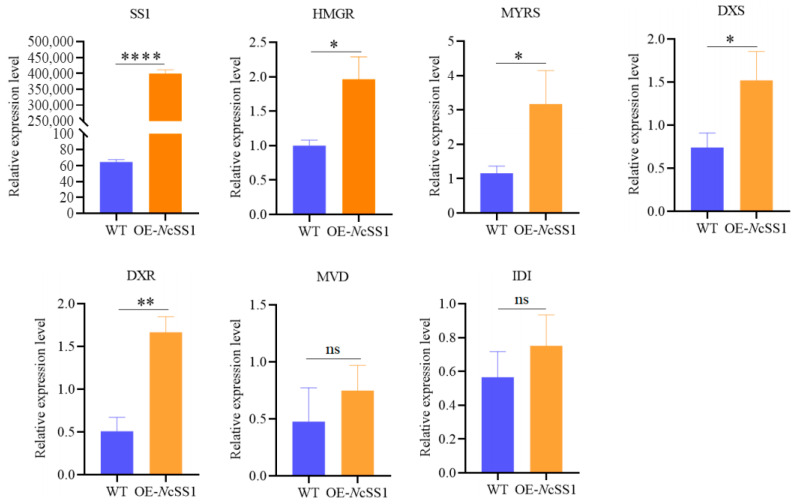
qRT-PCR Analysis of Some Genes Related to Metabolic Pathways of Terpenoids in Tobacco Leaves. Note: * *p* < 0.05, ** *p* < 0.01, **** *p* < 0.0001 and “ns” represents no significant difference.

Subsequently, using EF1α as the reference gene, qRT-PCR was performed on tobacco leaves transiently overexpressing *NcSS1*. The results showed that the relative expression level of *NcSS1* was substantially higher than that in the wild-type control, indicating strong expression of the *NcSS1* gene in tobacco leaves. In addition, we screened 15 regulatory genes potentially involved in the terpenoid metabolic pathway and conducted quantitative analysis. Most of the genes exhibited expression levels below the detection threshold. Among the detectable genes, *HMGR* (*NPaulChr06BG335390*, encoding 3-hydroxy-3-methylglutaryl-coenzyme A reductase), *MYRS* (*NPaulChr11AG613570*, encoding myrcene synthase), *DXS* (*NPaulChr05DG311290*, encoding 1-deoxy-d-xylulose-5-phosphate synthase), and *DXR* (*NPaulChr09AG482720*, encoding 1-deoxy-d-xylulose-5-phosphate reductoisomerase) showed significantly higher relative expression levels in *NcSS1*-overexpressing tobacco leaves compared with the wild-type control. Although the relative expression levels of *MVD* (*NPaulChr01DG042640*, encoding diphosphomevalonate decarboxylase) and *IDI* (*NPaulChr03AG150350*, encoding isopentenyl pyrophosphate isomerase I) did not reach statistical significance, their overall expression was notably elevated relative to the wild-type control (Figure 8).

## 3. Discussion

### 3.1. Volatile Profiling in Nymphaea Floral Scent

Floral fragrance constitutes a critical functional trait in plants, playing pivotal roles in growth, development, and evolutionary processes. For waterlilies, scent emission represents a key quality attribute directly influencing ornamental appeal and commercial value. Tropical waterlilies, renowned for their vibrant pigmentation and intense fragrance, hold significant economic importance in industrial applications and horticulture. This investigation employed integrated HS-SPME-GC-MS and RNA-seq analyses to characterize VOCs in stamens of tropical *N*. ‘Paul Stetson’ across three developmental stages. We identified 671 distinct VOCs and delineated the principal aroma-active constituents. Furthermore, key functional genes potentially regulating fragrance biosynthesis were elucidated. These findings provide valuable insights into the dynamic emission patterns of waterlily floral scent and establish a foundation for future breeding initiatives targeting fragrance enhancement.

Floral volatile profiles exhibit inherent complexity influenced by flowering phenology [40]. In this study, VOC diversity and abundance progressively increased during stamen development, peaking at anthesis (stage St3)—temporally aligned with the peak pollination window (Supplementary Table S1). Research indicates floral scent critically mediates pollinator attraction and reproductive success in waterlilies [32]. Although protogynous dichogamy characterizes most *Nymphaea* cultivars (pistil receptivity at anthesis versus stamen dehiscence on day 2), intense fragrance emission at anthesis enhances pollination assurance by attracting vectors when pistils are receptive. A proposed mechanism suggests VOC precursors accumulate during bud development, undergoing enzymatic conversion to volatile forms upon flower opening [41,42], potentially explaining elevated VOC levels in *N*. ‘Paul Stetson’ at full bloom. Notably, the quantity of aroma-active volatiles (rOAV >1) remained comparable between St2 and St3 across all chemical classes (Supplementary Tables S5 and S6). This indicates that enhanced accumulation of signature scent volatiles, rather than novel compound emergence, drives the intensified fragrance perception at St3.

*Nymphaea* species, representing basal angiosperms, serve as key models for investigating early co-evolution between floral traits and pollination vectors. As diurnal summer bloomers, tropical waterlilies typically anthesize around 08:00, synchronizing with bee foraging activity while avoiding peak thermal stress. In *N*. ‘Paul Stetson’ stage St3 stamens, we documented substantial terpenoid accumulation, notably α-farnesene and (E)-β-farnesene peaking at anthesis but undetectable at St1. Terpenoids—particularly sesquiterpenes like α-farnesene—function as dual-purpose semiochemicals: attracting pollinators while deterring herbivores [43,44,45]. Thus, farnesene enrichment at anthesis potentially enhances pollinator attraction and pathogen defense [46]. Our identification of key aroma-active compounds ((E)-β-farnesene, benzyl alcohol, humulene, benzyl acetate) aligns with reported scent profiles of diurnal tropical waterlilies [28,29,30,31]. In contrast, the floral scent profiles of tropical night-blooming waterlilies are predominantly characterized by aromatic ethers, aliphatic esters, and C5-branched chain esters. These species are primarily pollinated by cyclocephaline scarabs (Scarabaeidae, Cyclocephalini), a pattern that differs markedly from that of diurnal waterlilies in terms of both scent composition and pollinator guild [32]. These findings strongly suggest that the volatile blends emitted by waterlilies are closely associated with the specific pollinator taxa and their foraging behaviors.

### 3.2. The Unique Profile of Nymphaea Aroma

Floral scents comprise complex blends of VOCs exhibiting species-specific compositional signatures. Despite morphological similarities in floral structures, scent profiles remain chemically distinct [47,48]. Terpenoids represent the most diverse VOC class across plant taxa, critically shaping olfactory phenotypes [49]. Illustrative examples include: *Zingiber mioga*: dominated by β-guaiene, β-farnesene, δ-cadinene, and citronellyl isobutanoate (imparting woody/sweet notes) [49]; *Lilium* spp.: characterized by linalool, (E)-β-ocimene, and myrcene [50,51]; *Narcissus*: exhibits ocimene as the primary terpenoid despite inter-specific scent variations [50,51,52]; *Osmanthus fragrans*: features ionones and linalool as predominant terpenes [53,54]; *Nelumbo nucifera*: driven by 1,6-cyclodecadiene (1-methyl-5-methylene), α-ionone, (+)-δ-cadinene, and dl-isoborneol [23].

In *N*. ‘Paul Stetson’, the scent profile integrates terpenoids, esters, heterocyclic compounds, aldehydes, ketones, and alcohols, generating distinctive sweet/fruity/floral/woody sensory attributes (Figure 3A). Among 671 identified VOCs, 160 terpenoids constituted the principal olfactory contributors. Combined abundance and rOAV analyses identified five signature sesquiterpenes at anthesis (St3): humulene, (E)-β-farnesene, β-ionone, α-farnesene, and cis-2-methyl-5-(prop-1-en-2-yl)cyclohex-2-en-1-ol. At stage St3, integrated abundance and rOAV analyses identified critical aroma-active compounds beyond terpenoids, including: esters (geranyl isobutyrate and tetrahydro-6-pentyl-2H-pyran-2-one), heterocyclic compounds (2-methoxy-3-(2-methylpropyl)pyrazine and 6-pentyl-2H-pyran-2-one), aldehydes ((E)-2-dodecenal), ketones ((E)-6,10-dimethyl-5,9-undecadien-2-one), and alcohols (benzyl alcohol). These volatiles critically enhance fragrance emission and define the characteristic olfactory profile of waterlily. Notably, five compounds exhibited exceptional odor potency (rOAV >1000) with stable accumulation levels, while 6-pentyl-2H-pyran-2-one demonstrated rOAV > 600. Collectively, they impart distinctive fresh, green, creamy, and coconut sensory attributes, key determinants of waterlily’s unique floral signature. This chemosensory distinctiveness markedly differentiates *Nymphaea* scent from that of *Lilium* [50] and *Camellia* [55].

### 3.3. Molecular Mechanisms Underlying Floral Volatile Formation in Nymphaea

Integrated multiomics approaches have become indispensable for deciphering plant secondary metabolism, enabling comprehensive biological insights. Here, we employed transcriptomic and metabolomic strategies to investigate volatile compound composition and underlying regulatory mechanisms during stamen development in waterlily. Our analysis specifically targeted terpenoid metabolic regulation. Terpenoid biosynthesis primarily occurs via the plastidial MEP pathway and MVA pathway. These routes exhibit metabolic crosstalk rather than operating independently [48]. Transcriptome profiling across three developmental stages identified 47,951 DEGs in pairwise comparisons, providing a critical resource for elucidating fragrance biosynthesis. Among these, 179 terpenoid-related DEGs were annotated (Supplementary Table S9). Key terpenoid biosynthetic genes demonstrated stage-specific expression maxima at St3, including *NPaulChr03AG150350* (isopentenyl diphosphate isomerase, IDI1), *NPaulChr03BG162500* (geranylgeranyl diphosphate synthase, GGPPS), *NPaulChr06BG335390* (3-hydroxy-3-methylglutaryl-CoA reductase 1, HMGR1; MVA pathway), and *NPaulChr05DG311290* (1-deoxy-D-xylulose-5-phosphate synthase, DXS; MEP pathway). Previous studies indicate spatial and temporal congruence between floral scent biosynthetic gene expression and volatile accumulation [56,57]. We propose that elevated volatile production at St3 likely correlates with heightened expression of terpenoid pathway genes (e.g., HMGR, DXS), consistent with tissue-specific transcriptomic patterns in *N*. ‘Paul Stetson’ reported by Mao et al. [58] (Supplementary Table S8).

Metabolomic profiling identified farnesene as a signature aroma compound in *N*. ‘Paul Stetson’ (Supplementary Tables S6 and S12). We identified five candidate genes with elevated expression in St3 stamens potentially regulating fragrance biosynthesis. Among these, *NPaulChr13DG768090* and *NPaulChr13AG727020* encode 3S,6E-nerolidol synthase (NES1), though no nerolidol was detected in floral volatiles (Supplementary Table S1). Given evidence that NES1 catalyzes formation of α-farnesene, β-farnesene, and (E)-nerolidol from FPP [59] we postulate nerolidol undergoes farnesylation during *N*. ‘Paul Stetson’ scent emission [58]. Concurrent upregulation of *NPaulChr09AG491360* (farnesyl-diphosphate farnesyltransferase, SS1) and GGPPS-encoding genes (*NPaulChr03BG162500*, *NPaulChr03DG200280*, *NPaulChr04BG229240*) at St3 likely drives enhanced farnesene accumulation.

Furthermore, subcellular localization analysis was performed on the identified SS1 (*NPaulChr09AG491360*) and P450 (*NPaulChr07BG398620*), confirming that they function in the cytoplasm. This result is consistent with the prediction from the online tool WOLF PSORT (https://www.genscript.com/wolf-psort.html, accessed on 1 December 2025), which also localized both proteins to the endoplasmic reticulum within the cytoplasm. Transient overexpression of SS1 in tobacco leaves was conducted, and qRT PCR analysis of genes involved in terpenoid metabolic pathways revealed that the expression levels of several related genes were significantly higher than those in the wild-type control. These findings suggest that SS1 is likely involved in the biosynthesis of terpenoid compounds in waterlily. However, further experimental validation—such as through gene silencing techniques and stable genetic transformation—is required to confirm its functional role.

Notably, benzyl acetate exhibited maximal abundance and rOAV (>70) at St3 (Table S6), confirming its significant contribution to the scent profile. Two cytochrome P450 genes (*NPaulChr07BG398620*, *NPaulChr07DG426600*) were concurrently identified. Studies in *Prunus mume* suggest cytochrome P450s modulate benzyl acetate biosynthesis via precursors like benzaldehyde/benzyl alcohol [8]. Benzyl alcohol—a major aroma component in *N*. ‘Paul Stetson’—showed parallel accumulation dynamics, potentially providing substrate flux for benzyl acetate production (Supplementary Tables S6 and S12). Floral development and precursor accumulation coordinately regulate phenylpropanoid/benzenoid biosynthesis—the second most abundant class of floral volatiles [1]. In *N*. ‘Paul Stetson’, key aroma constituents include not only terpenoids but also phenylpropanoid/benzenoid compounds such as benzyl alcohol and benzyl acetate (Supplementary Tables S5 and S12). Transcriptomic analysis revealed 68 differentially expressed genes (DEGs) associated with phenylpropanoid metabolism across three developmental stages (Figure S6, Supplementary Table S10). Notably, several DEGs exhibited contrasting expression patterns, including *NPaulChr05DG311710* (encoding C4H homolog), *NPaulChr03BG159840* (encoding CCoAOMT homolog), and *NPaulChr12BG685880* (encoding CCR homolog), which showed peak expression at St1, while they demonstrated transcriptional downregulation by St3. We hypothesize that they primarily regulate non-volatile metabolic products (e.g., lignin biosynthesis) rather than volatile phenylpropanoid formation in stamens [60]. Phenylpropanoid/benzenoid pathways in waterlily volatile metabolism remain understudied. Further investigation of these gene families may elucidate benzyl alcohol/benzyl acetate biosynthesis and reveal their contributions to the characteristic floral fragrance.

Beyond terpenoids, esters, aldehydes, and ketones also constitute major odor-active constituents within the floral scent profile of waterlilies (*Nymphaea* L.). Each of these compound classes follows distinct biosynthetic pathways, governed by specific genetic regulatory networks that orchestrate the production of key volatiles. Future research, while continuing to advance terpenoid studies, should prioritize elucidating the biosynthetic routes of other critical aroma compounds and investigating the potential coordinated regulation of structural genes across these pathways. Furthermore, significant compositional differences exist in floral volatile profiles among *Nymphaea* species, particularly between diurnal and nocturnal bloomers. The underlying metabolic divergences in volatile synthesis and their adaptive evolutionary mechanisms warrant further investigation. Additionally, research into the post-transcriptional regulation and epigenetic modifications governing floral VOC biosynthesis in waterlilies remains largely unexplored, especially in comparison to other ornamental flowers. Elucidating these aspects will significantly deepen our understanding of aroma formation in waterlilies. This knowledge will provide a molecular foundation for the future genetic improvement of floral scent traits, ultimately enhancing both the ornamental merit and commercial utility of these plants.

## 4. Conclusions

**Figure 9:**
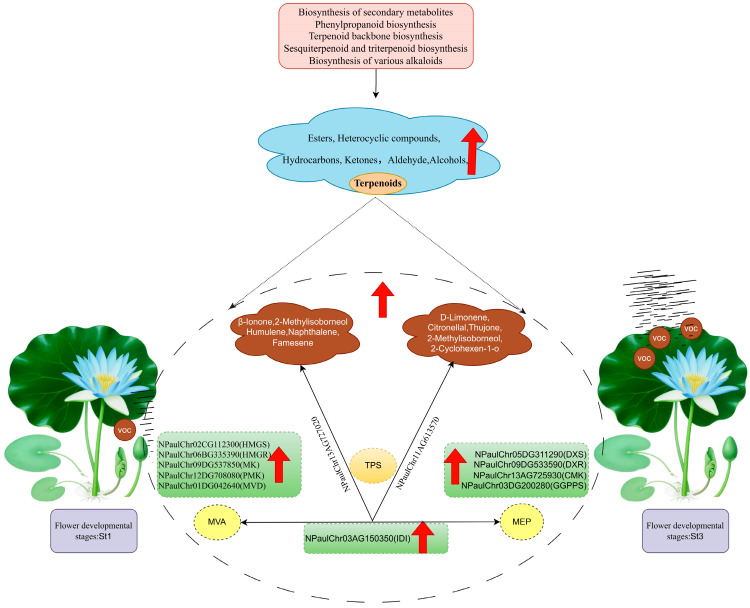
Diagrammatic overview of floral volatile emission dynamics in *Nymphaea* ‘Paul Stetson’ throughout flowering. During bloom development, VOCs exhibit substantial accumulation, encompassing terpenoids, esters, ketones, hydrocarbons, and aldehydes. Individual volatiles contributing to the characteristic scent profile are annotated. Putative structural genes associated with terpenoid biosynthesis via the MEP and MVA pathways—serving as primary drivers of these aromas in waterlily—are delineated. Red arrows denote upregulated expression of VOCs and structural genes during flowering.

Integrative transcriptomic and volatile metabolomic analyses identified the St3 stage as the critical period for volatile emission in waterlily. VOC formation was associated with pathways including secondary metabolite, phenylpropanoid, and terpenoid biosynthesis. Detected VOCs were predominantly terpenoids, esters, heterocycles, ketones, aldehydes, and alcohols, most peaking at St3. Correlation analysis identified structural genes in the MEP and MVA pathways and pinpointed five candidate genes potentially involved in floral fragrance regulation (Figure 9). These findings deepen our understanding of waterlily floral scent biosynthesis and provide a valuable resource for future genetic improvement of fragrance traits.

## 5. Materials and Methods

### 5.1. Plant Materials

*Nymphaea* ‘Paul Stetson’ were cultivated in the waterlily germplasm repository at the Flower Research Institute, Guangxi Academy of Agricultural Sciences, Nanning City, Guangxi Province, China. Building upon the six developmental stages defined by Wu et al. [61] from bud to the first day of flowering, and based on our multiyear practical observations, stamens from buds/flowers at three distinct developmental stages—S3, S5, and S6—were selected for this study. Specifically, St1, St2, and St3 represent stamens at the small flower bud stage, large flower bud stage, and the first day of anthesis, respectively (Figure 1A). All samples were collected in July 2024. Immediately after collection, stamens corresponding to each stage were designated St1, St2, and St3, respectively, flash-frozen in liquid nitrogen, and stored at −80 °C until analysis. *Nicotiana benthamiana* was used for transient expression and subcellular localization experiments.

### 5.2. Sample Preparation and Volatile Compounds Detected by HS-SPME-GC–MS

Sample processing and analytical procedures followed modified protocols established by Chen et al. [23]. Stamens representing three developmental stages were retrieved from −80 °C storage and cryogenically pulverized in liquid nitrogen. After thorough vortex homogenization, precisely 500 mg of each sample was portioned into headspace vials. Each aliquot received saturated NaCl solution and 10 µL of internal standard solution (50 µg/mL, BioBioPha/Sigma-Aldrich, Shanghai, China). VOCs were extracted using automated HS-SPME (SPME Arrow; CTC Analytics AG, Zwingen, Switzerland) prior to GC-MS (8890-7000D; Agilent, Shanghai, China) analysis.

HS-SPME parameters: (1) Sample equilibration: 60 °C for 5 min with agitation. (2) Extraction fiber: 120 µm DVB/CWR/PDMS. (3) Headspace exposure: 15 min at 60 °C. (4) GC inlet desorption: 250 °C for 5 min. (5) Fiber conditioning: 250 °C for 5 min (dedicated station).

GC configuration: (1) Column: DB-5MS capillary (30 m × 0.25 mm × 0.25 µm; Agilent). (2) Carrier gas: high-purity helium (≥99.999%) at 1.2 mL/min constant flow. (3) Injector: 250 °C (splitless mode) with 3.5 min solvent exclusion. (4) Temperature program: 40 °C (hold 3.5 min), 10 °C/min → 100 °C, 7 °C/min → 180 °C, 25 °C/min → 280 °C (hold 5 min).

MS operating conditions:.An electron ionization (EI) source was operated at 230 °C, with the quadrupole set to 150 °C and the transfer line maintained at 280 °C. Electron energy was 70 eV, and analytes were monitored in selected ion monitoring (SIM) mode, with specific qualitative and quantitative ions scanned for accurate detection.

Metabolite identification and quantification: Metabolite identification was performed by matching experimental data against a proprietary in-house database. For each sample group, all target ions were analyzed according to their elution order using time-segmented detection modes. A metabolite was positively identified when its detected retention time aligned with that of the standard reference, and all selected ions were present in the background-subtracted sample mass spectrum. For quantification, target ion peaks were selected, integrated, and calibrated to enhance accuracy. Raw data generated from mass spectrometry were processed using MassHunter 4.2 software for subsequent qualitative and quantitative analyses. Technical support was provided by MetWare Biotechnology Co., Ltd. (Wuhan, China).

### 5.3. Selection of Differentially Accumulated VOCs

Differential metabolites were accurately identified by employing both univariate and multivariate statistical approaches and by examining the data characteristics from multiple perspectives. Univariate statistical methods included hypothesis testing and fold-change (FC) analysis. Multivariate statistical techniques comprised principal component analysis (PCA) and orthogonal partial least squares discriminant analysis (OPLS-DA). Based on the variable importance in projection (VIP) scores derived from the OPLS-DA model (with at least three biological replicates), metabolites exhibiting differences between varieties or tissues were preliminarily screened. These candidates were further refined by integrating *p*-values or false discovery rates (FDRs) from univariate analysis (with at least two biological replicates) or by applying FC thresholds. The criteria for selecting significantly differential metabolites in this project were as follows: metabolites with VIP > 1, or with fold change ≥2 or ≤0.5, were considered statistically significant.

### 5.4. RNA Extraction and RNA-Seq Analysis

Total RNA was isolated from waterlily stamens using the FastPure Universal Plant Total RNA Isolation Kit (Vazyme Biotech Co., Ltd., Nanjing, China). RNA concentration was quantified with high precision using a Qubit 4.0 Fluorometer/MD Microplate Reader (Thermo Fisher Scientific, CA, USA). RNA integrity was assessed using a Qsep400 Bioanalyzer (Bioptic, Suzhou, China). Following quality control, mRNA was enriched from qualified RNA samples using oligo (dT) magnetic beads. The enriched mRNA was then fragmented using fragmentation buffer. First-strand cDNA synthesis was performed on the fragmented mRNA using random hexamer primers, followed by second-strand synthesis to generate double-stranded cDNA (ds cDNA). The ds cDNA was purified using DNA purification magnetic beads. The purified ds cDNA underwent end repair, A-tailing, and adapter ligation. Size selection of the adapter-ligated fragments was subsequently performed using DNA purification beads. Finally, the library was amplified via PCR. After library construction, quality assessment was conducted. Libraries meeting the quality thresholds were sequenced on an Illumina platform at Metware Biotechnology Co. Raw sequencing data were processed by removing reads containing adapter sequences, poly-N sequences, and low-quality reads. The resulting clean reads were then mapped to the *Nymphaea* ‘Paul Stetson’ reference genome (de novo sequenced and assembled by our research group, unpublished data) [62,63,64].

### 5.5. Identification of Differentially Expressed Genes (DEGs)

Transcript abundance was quantified using RSEM to obtain read counts mapped to each transcript. These counts were subsequently normalized and expressed as fragments per kilobase of transcript per million fragments mapped (FPKM) values [65]. Based on the alignment results and gene positional information within the reference genome, read counts per gene were summarized. Differential expression analysis was performed using DESeq2 (version 1.26.0) [66]. Genes exhibiting an absolute log2 fold change (|log2FC|) ≥ 1 and an adjusted *p*-value (false discovery rate, FDR) < 0.05 were designated as significantly differentially expressed genes [67]. Functional enrichment analysis of Gene Ontology (GO) terms and KEGG pathways (http://www.kegg.jp/kegg/pathway.html, accessed on 15 August 2024) for the DEGs was conducted using the clusterProfiler package (version 3.14.3) [68].

### 5.6. qRT-PCR Analysis

Qualified RNA samples were reverse-transcribed using the Hifair^®^ 1st Strand cDNA Synthesis Kit (Yeasen Biotechnology Co., Ltd., Shanghai, China). Synthesized cDNA underwent 10-fold dilution with RNase-free water and storage at −20 °C. Quantitative PCR amplification was performed in 20 μL reactions through 40 cycles on a LightCycler^®^ 480 II system (Roche Diagnostics, Basel, Switzerland) following manufacturer protocols. *ACT11* served as the endogenous reference [69]. Thirteen candidate DEGs implicated in floral scent metabolism were selected for validation. Relative expression levels were determined using the 2^−∆∆Ct^ calculation method. All qRT-PCR primer sequences used are listed in Supplementary Table S13.

### 5.7. Subcellular Localization and Tobacco Genetic Transformation

Using cDNA from stamens at stage St3 of *Nymphaea* ‘Paul Stetson’ as a template, specific primers were designed to amplify the coding sequence (CDS) region of the target gene excluding the stop codon. The resulting linearized gene fragment was digested and ligated via seamless cloning into the doubly digested pCAMBIA1301-GFP vector, generating a fusion construct of the target gene with GFP. The horizontal lines indicate the enzyme cleavage sites and, following digestion, cloning, and plasmid extraction, the resultant plasmid was introduced into Agrobacterium tumefaciens competent cells (strain GV3101). After confirming successful transformation, the bacterial suspension was infiltrated into tobacco leaves. Leaves infiltrated with Agrobacterium harboring the empty pCAMBIA1301-GFP vector (without the target gene) served as a negative control. The primer sequences for the recombinant vector are provided in Supplementary Table S14. All transgenic tobacco plants were kept in the dark at room temperature for 2 days before observation, then returned to normal growth conditions for 1 day prior to sampling for RNA extraction and qRT-PCR analysis. qRT-PCR primer sequences used are listed in Supplementary Table S13.

### 5.8. Statistical Analysis

Data processing and visualization were implemented in Excel 2023 (Microsoft), including mean and standard deviation computations. Primer design for qPCR utilized Primer 6.0 (Premier Biosoft, CA, USA). Column graphs were generated with GraphPad Prism 8.0.2 (GraphPad Software). Radar plots and principal component analysis (PCA) were constructed using Origin 2024 (OriginLab). Differential volcano plots and correlation heatmaps were produced through Metware Cloud (https://cloud.metware.cn/, accessed on 16 August 2024) and TBtools-II [70].

## Data Availability

The original contributions presented in the study are publicly available. The raw RNA-seq data have been submitted to the SRA database under accession number PRJNA1303240, and they are also freely available at: https://www.ncbi.nlm.nih.gov/sra/PRJNA1303240 (accessed on 7 August 2025).

## Supplementary Materials

The following supporting information can be downloaded at: https://www.mdpi.com/article/10.3390/plants15030384/s1, Figure S1. Thermogram of VOC accumulation for 9 samples; Figure S2. K-mear plot of differential VOCs; Figure S3. Differential VOC Sensory Flavor Radar Chart; Figure S4. Developmental stage-specific expression patterns of 23 TPS genes in *Nymphaea* ‘Paul Stetson’ stamens; Figure S5. Tissue-specific expression profiles of the 23 TPS genes; Figure S6. Developmental expression patterns of phenylpropanoid biosynthesis-related genes in *Nymphaea* ‘Paul Stetson’ stamens.Table S1. All VOCs detected in 9 samples; Table S2. different type Statistics of VOCs in three-stage stamens; Table S3. Statistics on the content of different types of VOCs in three-stage stamens; Table S4. Comparison of the number of differential metabolites in different groups; Table S5. Volatile metabolite rOAV value; Table S6. Metabolite accumulation with rOAV greater than 1000; Table S7. RNA sequencing quality control data; Table S8. DEGs in the transcriptome; Table S9. DEGs in the terpene synthesis pathway; Table S10. DEGs in the Phenylpropanoids synthetic pathway. Table S11. WGCNA analysis of 5 core genes; Table S12. VOCs accumulation detected (internal standard method); Table S13. qRT-PCR primer; Table S14. Primer sequences for constructing the pCAMBIA1301 recombinant vector.
