# Supplementary material for: Integrated Volatile Metabolome and Transcriptome Analysis Provides Insights into Floral Aroma Biosynthesis in Waterlilies (Nymphaea L.)
Source: Plants (Basel). 2026 Jan 27;15(3):384. doi: 10.3390/plants15030384 (PMC12899119; doi:10.3390/plants15030384)
Supplement: Supplementary file 1 [file plants-15-00384-s001.zip › Supplementary Fig..pdf]

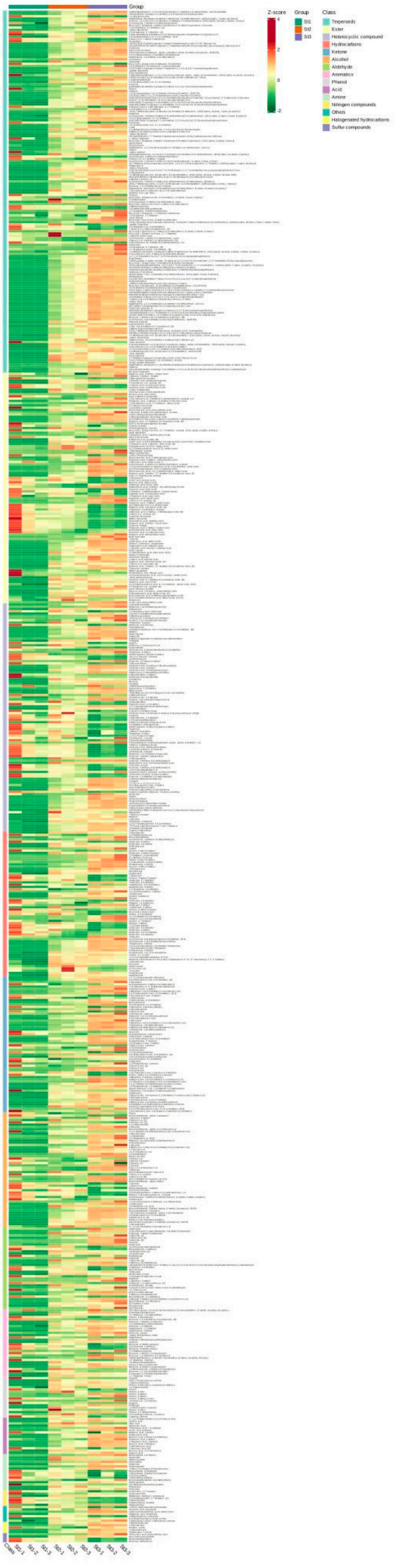

Figure S1 Thermogram of VOC accumulation for 9 samples

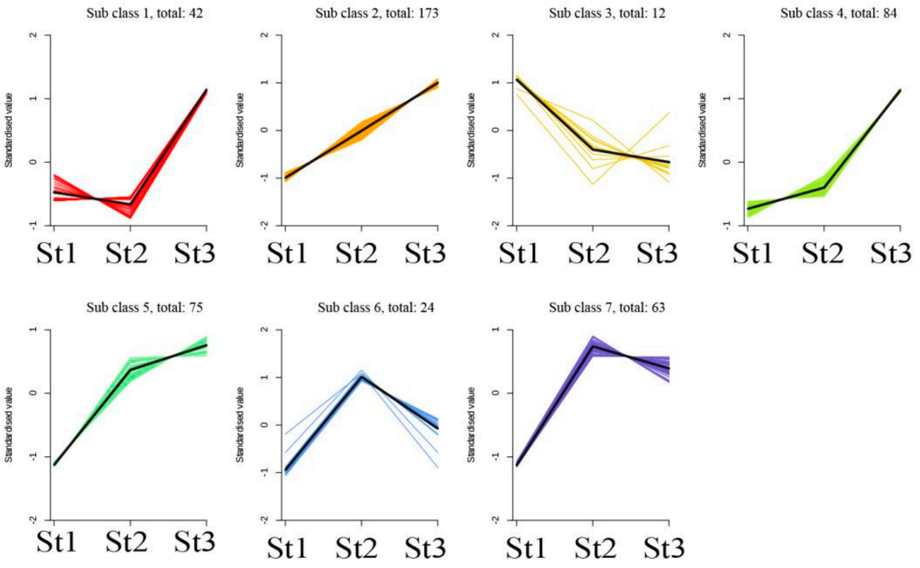

Figure S2 K-mear plot of differential VOCs

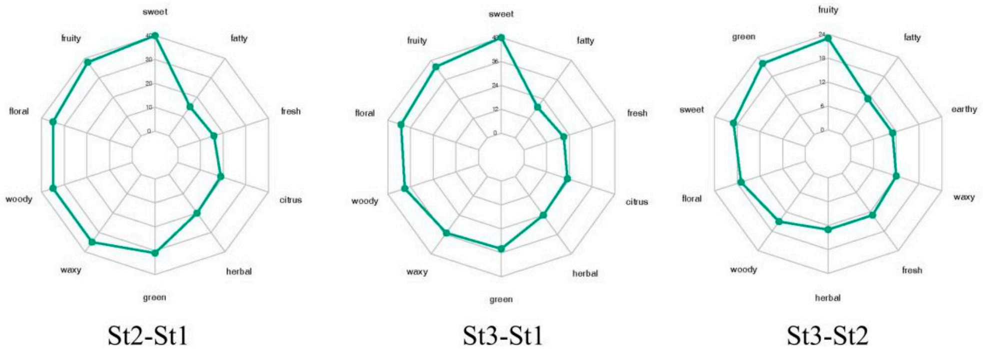

Figure S3 Differential VOC Sensory Flavor Radar Chart

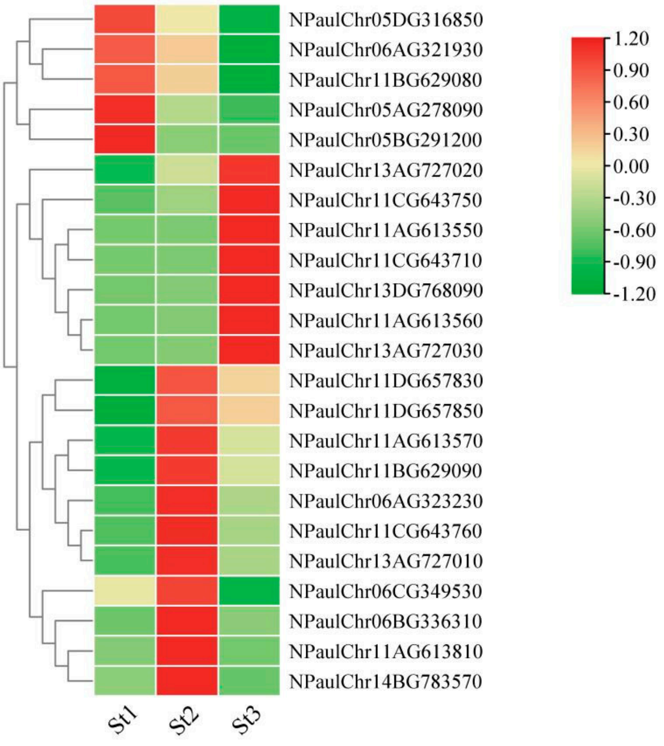

Figure S4 Developmental stage-specific expression patterns of 23 TPS genes in *Nymphaea* 'Paul Stetson' stamens

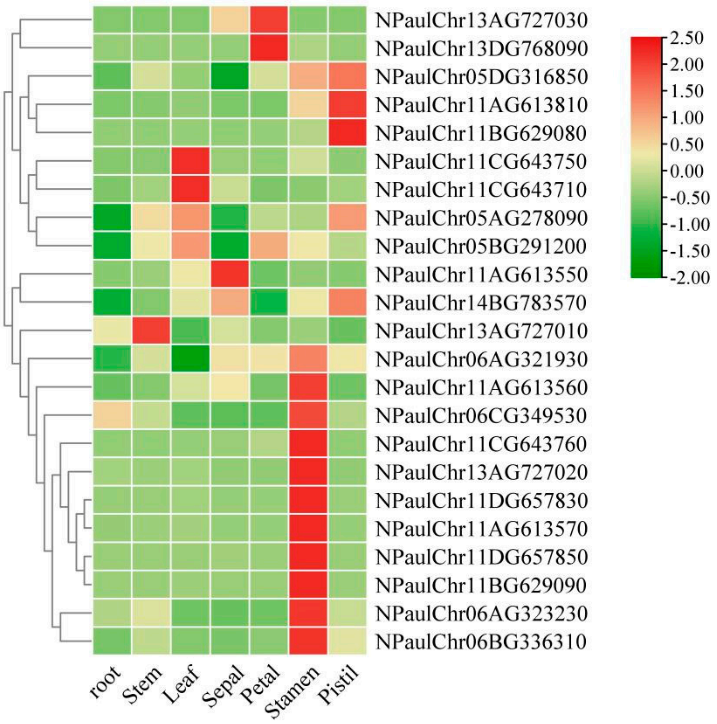

Figure S5 Tissue-specific expression profiles of the 23 TPS genes

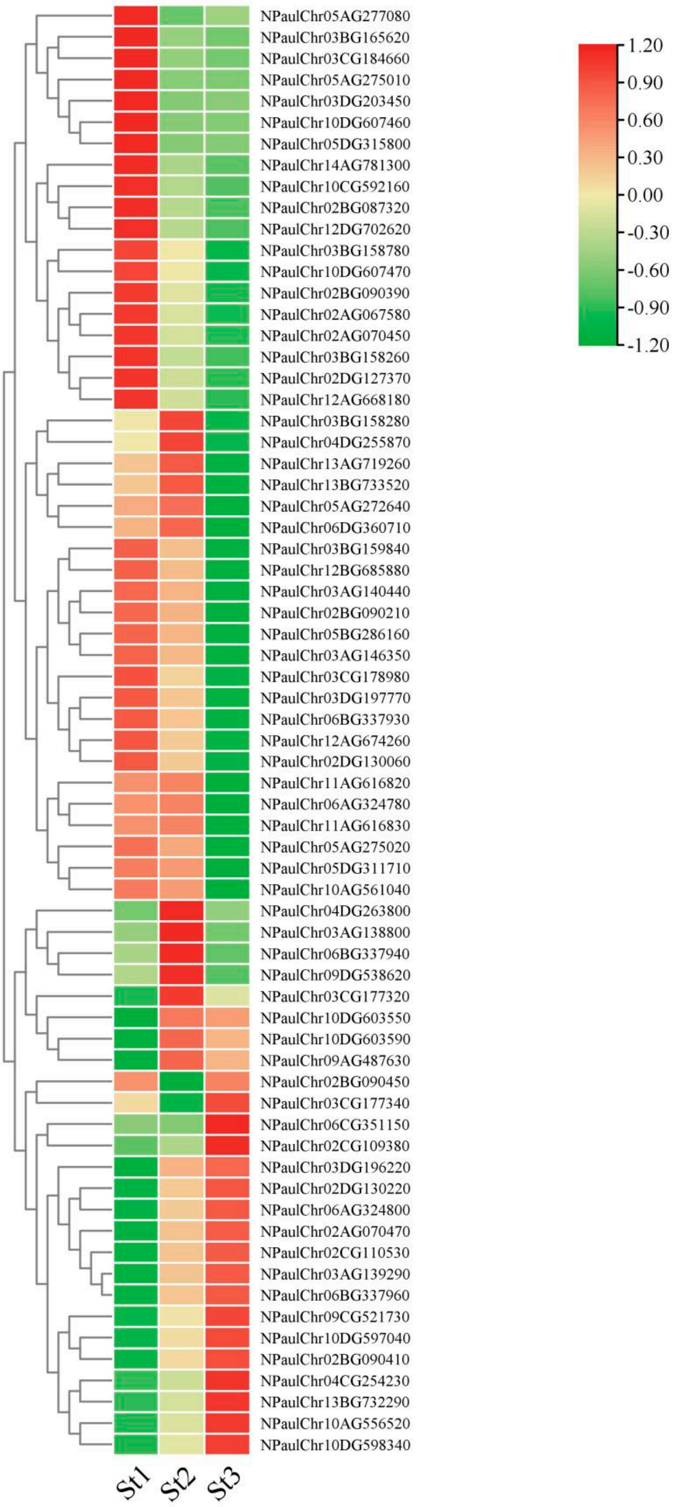

Figure S6 Developmental expression patterns of phenylpropanoid biosynthesis-related genes in *Nymphaea* 'Paul Stetson' stamens.
